# Intra-Erythrocyte Infusion of Dexamethasone Reduces Neurological Symptoms in Ataxia Teleangiectasia Patients: Results of a Phase 2 Trial

**DOI:** 10.1186/1750-1172-9-5

**Published:** 2014-01-09

**Authors:** Luciana Chessa, Vincenzo Leuzzi, Alessandro Plebani, Annarosa Soresina, Roberto Micheli, Daniela D’Agnano, Tullia Venturi, Anna Molinaro, Elisa Fazzi, Mirella Marini, Pierino Ferremi Leali, Isabella Quinti, Filomena Monica Cavaliere, Gabriella Girelli, Maria Cristina Pietrogrande, Andrea Finocchi, Stefano Tabolli, Damiano Abeni, Mauro Magnani

**Affiliations:** 1 Department of Clinical and Molecular Medicine, Sapienza Università di Roma, Via di Grottarossa 1035, 00189 Roma, Italy; 2 Department of Pediatrics and Child Neurology and Psychiatry, Sapienza Università di Roma, via dei Sabelli 108, 00185 Roma, Italy; 3 Department of Clinical and Experimental Sciences, Pediatrics Clinic and Institute of Molecular Medicine A. Nocivelli, Spedali Civili and University of Brescia, Piazza Spedali Civili, 1 25123 Brescia, Italy; 4 Unit of Child Neurology and Psychiatry, Spedali Civili and Università di Brescia, Piazza Spedali Civili, 1 25123 Brescia, Italy; 5 School in Reproductive and Developmental Science, Università di Trieste and Università di Brescia, Brescia, Piazzale Spedali Civili, 1 25123 Brescia, Italy; 6 Department of Molecular Medicine, Sapienza Università di Roma, Viale dell’Università 37, 00186 Roma, Italy; 7 Department of Pediatrics, Università di Milano, Fondazione IRCCS Ca’ Granda, via Commenda 9, 20122 Milano, Italy; 8 Department of Pediatrics, Ospedale Pediatrico Bambino Gesù and Università di Tor Vergata, Piazza di San Onofrio 4, 00165 Roma, Italy; 9 Istituto Dermopatico Immacolata, Via Monti Creta, 104, 00167 Roma, Italy; 10 Department of Biomolecular Sciences, Università di Urbino “Carlo Bo”, Via Saffi 2, 61029 Urbino, Italy and Erydel S.p.A, Via Sasso, 61029 Urbino, Italy

**Keywords:** Dexamethasone, Intra-Erythrocyte Dexamethasone, Ataxia Teleangiectasia, Ataxia Teleangiectasia Ataxia Treatment, Cerebellar Ataxia, ICARS, VABS

## Abstract

**Background:**

Ataxia Teleangiectasia [AT] is a rare neurodegenerative disease characterized by early onset ataxia, oculocutaneous teleangiectasias, immunodeficiency, recurrent infections, radiosensitivity and proneness to cancer. No therapies are available for this devastating disease. Recent observational studies in few patients showed beneficial effects of short term treatment with betamethasone. To avoid the characteristic side effects of long-term administration of steroids we developed a method for encapsulation of dexamethasone sodium phosphate (DSP) into autologous erythrocytes (EryDex) allowing slow release of dexamethasone for up to one month after dosing. Aims of the study were: the assessment of the effect of EryDex in improving neurological symptoms and adaptive behaviour of AT patients; the safety and tolerability of the therapy.

**Methods:**

Twenty two patients (F:M = 1; mean age 11.2 ± 3.5) with a confirmed diagnosis of AT and a preserved or partially supported gait were enrolled for the study. The subjects underwent for six months a monthly infusion of EryDex. Ataxia was assessed by the International Cooperative Ataxia Rating Scale (ICARS) and the adaptive behavior by Vineland Adaptive Behavior Scales (VABS). Clinical evaluations were performed at baseline and 1, 3, and 6 months.

**Results:**

An improvement in ICARS (reduction of the score) was detected in the intention-to-treat (ITT) population (n = 22; p = 0.02) as well as in patients completing the study (per protocol PP) (n = 18; p = 0.01), with a mean reduction of 4 points (ITT) or 5.2 points (PP). When compared to baseline, a significant improvement were also found in VABS (increase of the score) (p < 0.0001, ITT, RMANOVA), with statistically significant increases at 3 and 6 months (p < 0.0001). A large inter-patient variability in the incorporation of DSP into erythrocytes was observed, with an evident positive effect of higher infusion dose on ICARS score decline. Moreover a more marked improvement was found in less neurologically impaired patients. Finally, a 19 month-extension study involving a subgroup of patients suggested that Erydex treatment can possibly delay the natural progression of the disease.

EryDex was well tolerated; the most frequent side effects were common AT pathologies.

**Conclusions:**

EryDex treatment led to a significant improvement in neurological symptoms, without association with the typical steroid side effects.

**Trial registration:**

Current Controlled Trial 2010-022315-19SpA

## Background

Ataxia Teleangiectasia (AT) is a rare genetic disease [1,2] caused by biallelic mutations in the ataxia telangiectasia mutated (*ATM*) gene, most of which are truncations. The *ATM* gene encodes a PI3kinase, ATM, which plays a pivotal role in the control of cell cycle and DNA repair, targeting hundreds of substrates [3]. Homozygosity or compound heterozygosity for *ATM* mutations result in a multisystemic disorder, mainly involving nervous and immune system. AT patients with the classical phenotype present with early onset ataxia, oculocutaneous teleangiectasias, immunodeficiency, recurrent sinopulmonary infections, radiosensitivity, proneness to cancer and various neurodegenerative features. At cellular level a dramatic increase in spontaneous and radiation-induced chromosomal breaks is noticed. Neurologic features of AT include ataxia of the trunk and limbs, generally detected in the first years of life, progressive supranuclear ophthalmoplegia, dysarthria, swallowing incoordination, facial hypomimia and delayed peripheral neuropathy. Movement disorders such as dystonic postures and choreoathetosis could also be present and in some cases prevalent. In the classical form, patients are wheel-chair dependent by the age of ten [4], and their life expectancy is around twenty-five years [5].

Anecdotal reports and a few observational short term studies [6-9] suggested that betamethasone may be effective in improving neurological functions in AT patients. These observations were then supported by the results of a trial in which the patients assumed for a short-term oral betamethasone 0.1 mg/kg [10]. Most of the patients showed a clinical response, but side effects linked to the steroid use were observed.

A novel methodology for long-term delivery of low doses of steroids, based on the infusion of autologous erythrocytes loaded with dexamethasone sodium phosphate (DSP), has been developed [11]. Among corticosteroids dexamethasone is the most similar to betamethasone and its high anti-inflammatory potency, together with its lack of mineralcorticoid activity, make it a good choice for low-dose/long-term treatment as needed in chronic inflammatory diseases. DSP incorporated into erythrocytes is slowly converted by resident phosphatases to dexamethasone which is then released into the blood stream for about twenty-thirty days or longer.

Based on this evidence a multicenter, single-arm, open-label Phase II clinical trial was conducted in AT patients. The aim of the study was to evaluate the effect of DSP encapsulated into human autologous erythrocytes (EryDex), given as monthly infusions over a 6-month period, in improving neurological symptoms. Secondary objectives included the adaptive behaviour assessment and the safety and tolerability evaluations.

## Methods

### Patient population

A single-arm, open-label, 6-month Phase II study was performed to assess the effect of EryDex (dexamethasone sodium phosphate encapsulated in autologous erythrocytes; property of EryDel S.p.A., http://www.erydel.com) on neurological symptoms of AT patients enrolled in two centers in Italy (Department of Pediatrics and Child Neurology and Psychiatry, Sapienza University, Roma, and Department of Pediatrics, Brescia; under coordination of L.C.). Enrolling criteria included: age 3 years or older, classical AT phenotype (ataxic gait, oculomotor impairment, dysarthria, extrapyramidal disorders) with a preserved autonomous or partially supported gait; blood CD4+ lymphocytes counts/mm^3^ of ≥500 for patients aged 3–6 years or ≥200 for patients older than 6 years; weight over 15 kg; and proven molecular diagnosis of AT based on *ATM* gene mutations and/or ATM protein deficiency. Patients were excluded from the study on the basis of a history of severe impairment of the immunological system, current or previous neoplastic disease, chronic conditions representing a contraindication to the use of steroids, any serious concomitant systemic disorders that in the opinion of the Investigator would place the patient at excessive or unacceptable risk of toxicity, and any previous steroid use within 30 days before starting dexamethasone administration. The study was approved by the Ethics Committee of the involved clinical centers and all patients provided informed consent (along with consent of their parents or legal guardian, as required).

### Assessments

The primary objective of the trial was to evaluate the effect of EryDex in improving neurological symptoms of AT patients over a 6-month treatment period; this was assessed using the International Cooperative Ataxia Rating Scale (ICARS) [12-14]. The ICARS is a 100-point semi-quantitative scale including 19 items grouped into 4 multi-item subscales: Posture and Gait Disturbances (maximum score =34), Kinetic Functions (maximum score = 52), Speech Disorders (maximum score = 8), and Oculomotor Disorders (maximum score = 6). The ICARS rating was independently performed by 2 trained neurologists in each centre (VL and DD in Roma; RM and MA in Brescia) at baseline (before the first dose) and at 1, 3 and 6 months. Each evaluation was videotaped. Patient’s videotapes of each centre were independently and blindly reassessed by the neurologists of the other centre. The final score used for the statistical analysis remained that initially assigned by the centre-specific neurologists, due to the overall concordance among the different examiners. Adaptive abilities were assessed through Vineland Adaptive Behavior Scales (VABS), which was completed by the patients’ parents and explores strength and weakness of the patient in specific areas of adaptive behavior [15,16].

The safety profile of EryDex was assessed on the basis of the occurrence of treatment-emergent adverse events (TEAEs), including steroid-dependent adverse reactions and serious AEs (SAEs); on results from standard laboratory tests, physical examination, vital signs, ECGs; and on the need for concomitant medications and procedures. The following additional laboratory parameters were assessed: cholesterol (total, HDL, and LDL), glycosylated hemoglobin (HbA1c), CD4+ lymphocytes count, α–fetoprotein, and blood and urinary cortisol.

### Study design

**Figure 1:**
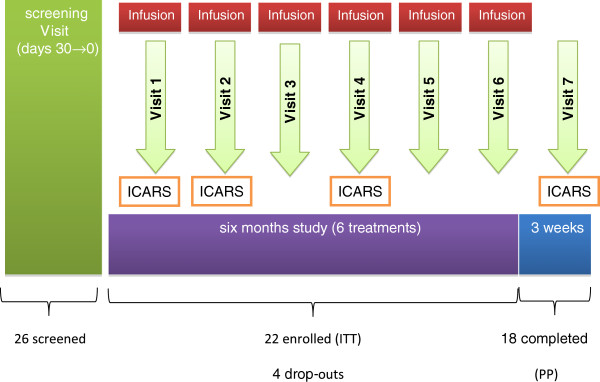
Study design.

The study consisted of a Screening Period (maximum duration 30 days), during which any possible prior corticosteroid treatment was withdrawn, followed by a 6-month Treatment Period (Figure 1). Screening assessments included medical history and demographics, physical and neurological examinations, vital signs, ECG and standard laboratory tests. Patients meeting all the selection criteria were enrolled in the treatment period and had the physical examination, vital signs and laboratory tests repeated, and all efficacy evaluations performed, at baseline (Day 0; Visit [V] 1) prior to receiving the first of 6 monthly (every 30 ± 10 days) infusions of EryDex.

The experimental treatment was prepared by collecting 50 ml of peripheral blood from the patient into a syringe containing heparin. Erythrocytes were loaded with DSP (2 vials of 250 mg DSP/10 ml added) using the EryDex System (http://www.erydel.com). The drug was entrapped and the cells resealed with the use of a “Red Cell Loader” device [11]. DSP-loaded red blood cells were then re-infused into the patient.

At each monthly visit physical examination, vital signs and routine laboratory tests were performed. The primary efficacy assessment (ICARS) was repeated at one month (V2); at the 3-month visit (V4) all efficacy assessments and the additional laboratory tests were performed before drug infusion. A final evaluation (V7), which included all efficacy and safety assessments, was done at 6 months after the initial infusion (one month after the last dose). The occurrence of any adverse event and the use of concomitant medication were recorded from the time of signing the consent form until the end of the study. An evaluation of the pharmacokinetic (PK) profile of EryDex was planned, with blood samples for PK analysis collected from three selected patients prior to dosing (0 hour), and 15 and 30 days post-dose at the first and fourth infusions.

### Statistical analyses

#### Primary endpoint

The primary analysis on ICARS was conducted on the intention-to-treat (ITT) population. A secondary analysis on ICARS was conducted on the Per Protocol (PP) Population. All secondary endpoints were analyzed both on the ITT and PP populations. The safety analysis was conducted on the Safety Population (SP). Changes at V2, V4 and V7 vs. V1 were analyzed with a Repeated Measures Analysis of Variance (RMANOVA) with possible covariates (among which were age and ICARS at baseline). Wilcoxon non-parametric tests between visits were also performed since overall analysis was significant, in order to help determine timing. A two-tailed α-level of *0.05* was considered statistically significant.

#### Secondary endpoints

**VABS** The scores were aggregated and for each of the four scales and each of the eleven subscales RMANOVA (or Friedman as appropriate) was performed.

#### Safety endpoints

Clinically significant changes of laboratory tests and vital signs have been summarized in shift-tables; the other safety endpoints were reported descriptively.

#### Special laboratory parameters

For each of the special laboratory parameters measured at V1, V4 and V7 absolute values and changes with respect to baseline were descriptively reported. A RMANOVA analysis with covariates was conducted on the absolute values, followed if significant by paired *t*-tests between visits.

#### Post-Hoc analyses (PP population)

A wide interindividual variation in concentrations of encapsulated dexamethasone in erythrocytes as well a wide individual change in final ICARS scores became evident after the conclusion of the study. Therefore, the ensuing supplementary analyses aimed to explore the relationship between the degree of clinical improvement and the efficiency of dexamethasone loading in erythrocyte was performed. Each patient was clustered according to: i) a arbitrary cutoff value of -10 point decrement in total ICARS scores at V7 relative to the baseline value (≥ -10 *responders*; ≤ -9 *non*-*responders*); and ii) a arbitrary cutoff of 5 mg/bag, as a measure of effective erythrocyte loading (≥5 loaders; ≤ 4.9 poor-loaders). Fisher’s exact test was used to detect significant difference among sub groups. A two-tailed α-level of *0.05* was considered statistically significant.

Finally the maximally sensitive ICARS subtotals were considered.

At the end of the trial 4 subjects (cases 02.05, 02.02, 02.08, and 02.01 in Additional file 1: Table S1; mean age 12.5 years; range 11–16.6) required and were authorized to continue Erydex treatment. So far they have been treated for an adjunctive 19-month period. The variations of ICARS score recorded in these patients during this extension study were compared with those detected in 4 AT patients matched for the ICARS score at the beginning of this extended study (cases 01.02, 01.07, 01.10, and 01.11 in Additional file 1: Table S1; mean age 14.4 years; range 10–21), who had discontinued the treatment as initially scheduled by the study design.

## Results

### Disposition

A total of 26 AT patients were screened and 22 were enrolled in the trial. Four AT patients had residual ATM protein amounts (10% to 50% of normal). The kinase assays (presence/absence of autophosphorylation of ATM in Serine1984 and phosphorylation of p53 in Serine15) demonstrated absence of kinase activity in these subjects. Thescreening failures were due to concomitant disease (one patient) and CD4+ lymphocytes count below the protocol cut-off (three patients). Of the 22 patients enrolled (Additional file 1: Table S1, ITT population), 16 received the six planned infusions of EryDex; 2 patients received five infusions completing the study (Per Protocol population) (Additional file 2: Table S2); 4 patients discontinued prematurely: one for withdrawal of consent, one for a protocol violation (CD4+ lymphocytes count below cut-off at baseline), and 2 for decrease in CD4+ lymphocytes count (Additional file 3: Figure S1). Two patients were on inhalational steroids at screening, and these medications were discontinued one month before starting the treatment.

*Demographics and Disease Characteristics of the ITT population at V1* (Additional file 1: Table S1).

The mean age of the AT patients was 11.2 (±3.5) years, with half (11 of 22) of the patients being males. All patients were Caucasian with a mean weight of 29.1 (±9.5) kg and BMI of 15.6 (±2.6). The average age of diagnosis of AT was 60.0 (±35.2) months, with a mean age of disease onset at 25.9 (±17.5) months. The mean baseline total scores for the ICARS and VABS were 50.6 (±12.8) and 5.5 (±2.0), respectively (Table 1 and Table 2). Mean baseline scores on the sub-scales of ICARS were as follows: posture/gait disturbance 20.9 (±7.1); kinetic functions 23.1 (±6.2); speech disorders 3.5 (±1.4); and oculomotor disorders 3.2 (±1.0).

**Table 1:** Mean ICARS Total Score and Changes from Baseline (V1) by Visit (ITT Population)

	**ICARS total score**	**95% CI for the mean**	**Mean**Δ **(SD)**	**95% CI for mean**Δ	**Median**	**Min-max**	p-value*
V1	50.6 (12.8)	(45.2/56)	-		52.5	8–72	-
V2	48.4 (11.4)	(43.6/53.2)	-2.2 (5.6)	(-4.6/0.2)	48.5	14–68	0.107
V4	47.4 (11.5)	(44/51.8)	-3.4 (7.3)	(-6.7/0.1)	49.0	14–62	0.054
V7	46.6 (12.3)	(41.4/51.8)	-4.0 (7.5)	(-7.1/-0.9)	48.0	14–62	*0.024*

*Comparison vs. baseline (V1) using Wilcoxon non-parametric test.

**Table 2:** Mean VABS Total Score by Visit (ITT Population)

	**Mean (SD)**	**95% CI for the mean**	**MeanΔ (SD)**	**95% CI for mean**Δ	**Median**	**Min-max**	p-value*
V1	5.5 (2.0)	(4.7/6.3)	-		5.1	2.1–11.0	-
V4	6.5 (2.1)	(6/7.8)	1.0 (0.9)	(0.7/1.5)	6.2	3.6–10.9	<*0.0001*
V7	6.9 (2.3)	(6/8)	1.3 (1.2)	(0.8/1.8)	6.9	2.5–11.1	<*0.0001*

*Comparison vs. baseline (V1) using Wilcoxon non-parametric test.

### Efficacy results

Results for the primary efficacy measure (change in total ICARS score) for the ITT population (n = 22) indicated an overall statistically significant (p = 0.02, RMANOVA) improvement from baseline with EryDex treatment. The average4-point decline in the total ICARS score observed at 6 months (V7), compared to baseline, was statistically significant (p = 0.024, Wilcoxon non-parametric test) (Table 1). An analysis performed on the Per Protocol population (n = 18) showed similar results, with statistically significant improvements at 3 and 6 months (p = 0.032 and p = 0.010, respectively) (Table 3). Once restricted the analysis to the 4 patients with lowest basal ICARS score (42 ± 1) (Additional file 2: Table S2), the mean improvement they experienced was -12.75points (see video recording included as Additional file 4). In the analysis of the individual ICARS sub-scales, the greatest overall improvement was seen in the “Kinetic Functions” sub-scale (*p* = *0.0017*, RMANOVA), with a statistically significant decrease in the score at 3 (V4) and 6 (V7) months (p = 0.027 and p = 0.003, respectively; ITT) (Table 4).

**Table 3:** Mean ICARS Total Score by Visit (PP Population)

	**Mean (SD)**	**Mean**Δ **(SD)**	**Median**	**Min-max**	p-value*
V1	51.8 (7.7)	-	52.5	37–68	-
V2	49.4 (8.5)	-2.4 (4.9)	48.5	36–68	0.059
V4	47.9 (9.0)	-3.9 (7.1)	49.0	30–61	*0.032*
V7	46.7 (10.1)	-5.2 (7.0)	46.0	26–61	*0.010*

*Comparison vs. baseline (V1) using Wilcoxon non-parametric test.

**Table 4:** Mean ICARS Kinetic Functions Subscale Score by Visit (ITT Population)

	**Mean (SD)**	**95% CI for the mean**	**Mean**Δ **(SD)**	**95% CI for mean**Δ	**Median**	**Min-max**	p-value*
V1	23.1 (6.2)	(20.5/25.7)	-		23.5	2–36	-
V2	22.0 (5.0)	(19.9/24.1)	-1.1 (3.3)	(-2.5/0.3)	22.0	6–32	-
V4	20.6 (5.2)	(19/22.6)	-2.5 (4.7)	(-4.6/-0.6)	21.0	6–28	*0.027*
V7	20.2 (5.1)	(18.1/22.3)	-2.9 (3.8)	(-4.5/-1.3)	20.5	6–28	*0.003*

*Comparison vs. baseline (V1) using Wilcoxon non-parametric test.

Significant improvements were noted in adaptive behavior, as assessed by the VABS total score (p < 0.0001, ITT, RMANOVA), with statistically significant increases at 3 and 6 months (p < 0.0001), compared to baseline (Table 2). Among the VABS sub-scales, all but “Free Time” and “Social Rules” showed statistically significant improvements.

### Pharmacokinetic profile

Due to the small amount of blood available, the samples were pooled together and dexametasone was found to be undetectable at pre-infusion and in the range 8–11 nM at time 15 and 9–10 nM at time 30 days post infusion.

### Supplementary analyses

Data analysis revealed: a) a wide interindividual variability in the variations of ICARS scores from basal to 7th assessment (range -16 +9); b) a substantial variability in the patient-specific DSP-erythrocyte loading [the mean (SD) dose of DSP measured in all the infusion bags for each patient ranged from 0.7 (0.1) to 18.6 (1.9) mg] (Additional file 2: Table S2); c) a greater proportion of loaders among females rather then males (80% vs 27%). Statistic analysis confirmed that the efficiency of the erythrocyte loading was related to greater improvement (Fisher’s exact test p = 0.0090) and consequently a greater proportion of responders among females (73%), compared to males (18%) (Fisher’s exact test p = 0,0015). Loaders had a mean percent decrease (improvement relative to baseline) of -14.4% for the ICARS total score, compared to -4.7% for “non-loaders”. Greater improvement in “loaders” vs. “non-loaders” was also observed for the ICARS Kinetic Functions sub-scale, even though the difference did not reach statistical significance (Fisher’s exact test p = 0.13).

A residual interindividual variability in response to treatment could not be ascribed to any of the considered variables as 4 out of 10 of loaders did not showed relevant improvement under treatment, while ICARS score declined of -11 points in 1 out of 10 of poor-loaders.

Finally, a mean 4.25 point-improvement (range +1 to -7) of ICARS score was detected after 19-month extension of Erydex treatment in subjects undergoing the extended study, while a mean 7 point-worsening (range +1 to +14) was found in ICARS matched controls (Additional file 5: Table S3 and Additional file 6: Table S4).

### Safety results

The Safety population included all 22 patients. Overall, 15 (68.2%) of patients experienced a total of 29 TEAEs, most of which were rated as mild (60% of patients) and not related to the study medication (>90% of patients). The most common TEAEs, occurring in 10% of patients, were cough, fever, flu syndrome and otitis, each occurring in 3 (14%) patients. Two TEAEs (pneumonia and bronchopneumonia) occurring in one patient were rated as severe. Two possible SAEs were reported in a 7-year old female (bronchopneumonia) and in an 11-year old male (bronchiectasis with bleeding), both of which required hospitalization. These events, which are common in AT patients and had occurred in the sample as a whole with a similar frequency as in the pre-treatment period. were not considered to be related to the study medication. The most frequently used concomitant medications were antibacterials and analgesics.

Only one patient experienced an AE judged to be related to the study medication, a mild increase in cholesterol levels (from 5.17 mmol/L at baseline to 5.66 mmol/L at final visit). Two patients had a >20% decrease in CD4+ lymphocytes count during the study period, which resulted in premature discontinuation. These were not reported as AEs by the Investigator, but were considered AEs as per protocol definition and rated as *mild* with *unsuspected* relationship to study medication.

There were no clinically meaningful changes in mean values for routine laboratory parameters with EryDex treatment, except for serum iron, which showed a mean decrease of about 20% at final assessment vs. baseline, with 8 patients having newly occurring abnormal low values at the final visit (one reported as a TEAE) (Additional file 7: Table S5). For the special laboratory parameters, no clinically meaningful changes were observed, except for urinary cortisol, which showed an approximately 26.3% decrease from baseline to the final visit (p = 0.016). Although this could indicate an effect of EryDex on the hypothalamo-pituitary-adrenal axis, a comparison of blood and urinary cortisol levels vs. dose did not indicate any relationship. No clinically significant changes with EryDex treatment were observed for vital signs, ECGs, or physical examination findings. No significant changes in blood pressure were detected in any patient during the entire trial (Additional file 8: Table S6).

## Discussion

In this open-label study involving AT patients aged more than 3 years the EryDex treatment for 6 months led to a significant improvement in ICARS, the primary efficacy measure which assesses the key symptoms of the disease. Significant benefits of EryDex treatment were more relevant in the Kinetic Functions sub-scale of the ICARS and in measures assessing the patients’ adaptive behavior (VABS).

A limiting issue in all the trials focusing on AT is the lack of a dedicated rating scales to assess the complex neurologic impairment in this disease and the variable contribution of different movement disorders to the morbidity of the patients. Waiting for an *ad hoc* scale for A-T, which is being currently validated [17], ICARS remains the most extensively used scale for children with ataxia. It is characterized by a good inter-rater reliability and it is designed for ataxic symptoms regardless of etiology [12]. Focusing on AT, main constraints of the scale are: the lack of a scoring subscale for dyskinetic movements (such as chorea, dystonia, athetosis, myoclonus), which are prevalent in a low percentage of AT subjects [18] and the non-tailored sub items dealing with eye movement impairment and speech disorders. VABS was used in our study to provide additional information on the global functioning in term of daily living skills. In patients with a more severe neurological impairment, VABS score seemed to reflect better than ICARS score the clinical improvement detected by the clinical observation. Few studies addressed the issue of therapeutic strategies in AT patients. Nissenkorn et al. [19] demonstrated that amantadine sulfate improves ataxia in AT patients with a mild impairment (ICARS 38.94 ± 10.13).

Recently, Zannolli et al. [10] performed a multicenter double-blind randomized short term (30 days) trial with betamethasone *vs* placebo in AT patients with a moderate neurological impairment (median initial ICARS total score 44). Even though patients under betamethasone experienced a significant reduction of ICARS score, they also experienced important adverse events (increases in BMI, cholesterol, and HDL cholesterol and decrease in blood phosphorus). In contrast, in this study EryDex treatment was well tolerated during a six month trial and no variations were observed in vital signs and biochemistry. The most common TEAEs were moderate and considered ‘not related’ to the study medication, being conditions generally related to the disease.

Our results are impressive taking in consideration that most of the patients had an advanced form of the disease (score at baseline: 50.7 ± 12.8, range 42 to 68). When the patients with milder neurological involvement were considered, the improvement was more remarkable, suggesting the efficacy of treatment as function of the stage of the disease. Moreover, the clinical improvement at ICARS scores was even more relevant in patients with the most efficient dexamethasone loading in the erythrocytes. As this point is concerned, a greater proportion of females, compared to males, had mean doses of DSP of 5 mg or more. Mean value (and SD) of DSP content in the processed erythrocytes was 10.39 ± 6.28 in female, and 4.79 ± 4.68 mg/bag in male patients (n = 10 and 11 respectively) and t-test showed statistical significance of the difference between the two groups (P < 0.03). Since a higher dose was shown to correlate with a greater response on ICARS, the proportion of female patients having a clinically meaningful response (i.e. >10 ICARS point improvement on the ICARS total score at the final visit) was higher than for males. One finding that needs further exploration is the low level of DSP-erythrocyte encapsulation (almost half of the patients had mean infusion bag doses of less than 5 mg of DSP) and high level of variability in this trial, in comparison to previous studies that used EryDex as treatment device [20-25]. This may be due to an intrinsic biochemical change in the cell membrane of the erythrocyte in AT patients, as previously reported [26,27]. These changes in physico-chemical properties could influence the encapsulation of DSP into AT patients’ erythrocytes and the consequent release of the active drug into the circulation. Further refinements in the procedure for loading erythrocytes with DSP are being made to ensure higher entrapment and lower intra- and inter-individual variability. No effect of age on the response to EryDex treatment was found.

This study provides support for the use of intra-erythrocyte DSP (EryDex) in the treatment of patients suffering AT, a so far untreatable disease. By continuously providing low dose levels of dexamethasone, EryDex should avoid the side effects of long-term steroid use, a great result in a population of patients which need a chronic treatment. The feasibility of a large double-blind, placebo-controlled trial should be carefully evaluated in order to confirm these preliminary findings and to assess long-term effectiveness and safety. Looking at future studies, we need to explore the efficacy of treatment in preventing or postponing the key milestones in the natural history of AT, so changing the disease course as already reported for other disorders [28]. From this point of view, as preliminary observation, the extended study performed in 4 subjects suggests that Erydex treatment could actually delay the natural progression of the disease.

It is worth noting that *in vitro* dexamethasone was shown to induce a non-canonical splicing that leads to translation of a short ATM variant retaining kinase activity. Thus, ATM may be restored by a new molecular mechanism which overcomes must of the mutations so far described in *ATM* gene [29]. Once confirmed in other studies this observation will provide the molecular basis for dexamethasone action in AT patients. Other mechanism(s) of dexamethasone that could contribute to the explanation of observed clinical improvement (i.e. dexamethasone anti-inflammatory effects) should be considered and are under investigation.

## Conclusions

DSP delivered by erythrocyte is a promising treatment strategy for long-term steroid administration in AT patients, which confirms the efficacy of the steroids in improving the neurological deficits associated with the disease while minimizes the side effects resulting from the oral administration of these drugs. The potential efficacy of this treatment in modifying the natural course of AT warrants further studies.

## Abbreviations

AT: Ataxia Teleangiectasia; ATM: Ataxia telangiectasia mutated; BMI: Body mass index; EryDex: Dexamethasone sodium phosphate encapsulated in autologous erythrocytes; ICARS: International Cooperative Ataxia Rating Scale; ITT: Intention-to-treat; PK: Pharmacokinetic; PP: Per Protocol population; SAE: Serious adverse effect; DSP: Dexamethasone sodium phosphate; RMANOVA: Repeated Measures Analysis of Variance; TEAE: Treatment-emergent adverse events; VABS: Vineland Adaptive Behavior Scales.

## Competing interests

MM hold stock ownership in EryDel S.p.A. No competing interests for all other authors.

## Authors’ contributions

All authors participated to the conception, design, analysis and interpretation of the content, manuscript preparation and revision and have read and approved the final version of the manuscript. All authors read and approved the final manuscript.

## Supplementary Material

Additional file 1: Table S1 Demographic data (ITT population). Click here for file

Additional file 2: Table S2 Number of treatments for patients (divided for Trial Center: 1–Roma, and 2-Brescia), mean DSP loading for patient, ICARS and VABS variation (ITT population). Click here for file

Additional file 3: Figure S1 Study disposition. Click here for file

Additional file 4 **Video Legend.** Basal and V7 neurological examinations in patient 01–01 showing improvement in ICARS subtests. Click here for file

Additional file 5: Table S3 ICARS and VABS scores in 4 patients treated for an adjunctive 19-month period as compared with 4 ICARS score matched controls. Click here for file

Additional file 6: Table S4 Values for Special Laboratory Parameters and adverse events in 4 patients treated for an adjunctive 19-month period as compared with 4 ICARS score matched controls. Click here for file

Additional file 7: Table S5 Changes from Baseline to the Final Visit for special laboratory parameters (ITT Population). Click here for file

Additional file 8: Table S6 Group data for Vital and Physical Signs at V1 and V7 (ITT Population). Click here for file
