# Supplementary material for: Intra-Erythrocyte Infusion of Dexamethasone Reduces Neurological Symptoms in Ataxia Teleangiectasia Patients: Results of a Phase 2 Trial
Source: Orphanet J Rare Dis. 2014 Jan 9;9:5. doi: 10.1186/1750-1172-9-5 (PMC3904207; doi:10.1186/1750-1172-9-5)
Supplement: Additional file 1: Table S1 — Demographic data (ITT population). [file 1750-1172-9-5-S1.docx]

Additional Table 1. Demographic data (ITT population) divided for Trial Center: 1–Roma and 2-Brescia.

| Patient ID | sex | age  (years/months) | *ATM* genotype | ATM protein |
| --- | --- | --- | --- | --- |
| Center 1 |  |  |  |  |
| 01-01 | F | 11/7 | n.d. | absent |
| 01-02 | F | 7/10 | n.d. | absent |
| 01-03 | F | 10/7 | 1898+1G>T / ? | 10% |
| 01-04 | F | 10/8 | 7517del4 / ? | absent |
| 01-05 | M | 10/1 | 717delCCTC / 717delCCTC | absent |
| 01-06 | F | 6/4 | 3894insT | absent |
| 01-07 | M | 10/2 | 97delC / 2113del T | absent |
| 01-08 | M | 12/3 | 4344-4345insA / IVS47-9G>A | n.d. |
| 01-09 | F | 6/10 | 126G>A / del-700Ex46+1406 | n.d. |
| 01-10 | F | 18/1 | 4396C>T / 9139C>T | absent |
| 01-11 | F | 18/9 | 3802delG / ? | absent |
| Center 2 |  |  |  |  |
| 02-01 | M | 8/8 | 6679C>T / 8484del A | 10% |
| 02-02 | M | 8/7 | 5979del5 / 7408T>G | 20% |
| 02-03 | M | 11/6 | 3111delT / 3576G>A | n.d. |
| 02-04 | F | 17/8 | 3111delT / 3576G>A | n.d. |
| 02-05 | M | 14/2 | IVS12+1G>T / 3576G>A | n.d. |
| 02-06 | M | 12/10 | R111X / L3035F | n.d. |
| 02-07 | M | 13/7 | 1369C>T / 3576G>A | absent |
| 02-08 | M | 10/2 | 6679C>T / 6679C>T | absent |
| 02-09 | M | 10/7 | 331+2T>G / ?4-20dup | absent |
| 02-10 | F | 3/3 | 5932G>T / 8278C>T | absent |
| 02-11 | F | 3/8 | c.3291delC / c.8977C>T | 50% |

n.d.= not done
