# Supplementary material for: Intra-Erythrocyte Infusion of Dexamethasone Reduces Neurological Symptoms in Ataxia Teleangiectasia Patients: Results of a Phase 2 Trial
Source: Orphanet J Rare Dis. 2014 Jan 9;9:5. doi: 10.1186/1750-1172-9-5 (PMC3904207; doi:10.1186/1750-1172-9-5)
Supplement: Additional file 2: Table S2 — Number of treatments for patients (divided for Trial Center: 1–Roma, and 2-Brescia), mean DSP loading for patient, ICARS and VABS variation (ITT population). [file 1750-1172-9-5-S2.doc]

Additional Table 2. Number of treatments for patients (divided for Trial Center: 1–Roma and 2-Brescia), mean DSP loading for patient, ICARS and VABS variation. (ITT population)

| Patient ID | Number of infusions | DSP mean dose (mg/bag) | ICARS V1* | ICARS V7* | ICARS  ∆1-7* | VABS  V1* | VABS  V7* | VABS  ∆1-7* |
| --- | --- | --- | --- | --- | --- | --- | --- | --- |
| Center 01 |  |  |  |  |  |  |  |  |
| 01-01 | 6 | 9.6 | 58 | 46 | 12 | 7.0 | 9.9 | 2.9 |
| 01-02 | 6 | 8.5 | 42 | 26 | 16 | 6.9 | 9.7 | 2.10 |
| 01-03 | 6 | 4.7 | 47 | 38 | 9 | 4.4 | 5.9 | 1.5 |
| 01-04 | 6 | 18.6 | 57 | 46 | 11 | 4.6 | 6.9 | 2.3 |
| 01-05 | 6 | 3.5 | 68 | 61 | 7 | 4.9 | 7.9 | 3 |
| 01-06 | 4 | 1.0 | 42 | ND | ND | 2.11 | ND | ND |
| 01-07 | 6 | 1.9 | 51 | 50 | 1 | 4.4 | 8.8 | 4.4 |
| 01-08 | 6 | 0.7 | 53 | 53 | 0 | 5.8 | 7 | 1.4 |
| 01-09 | 6 | 17.8 | 42 | 28 | 14 | 9.6 | 11.1 | 1.7 |
| 01-10 | 6 | 17.4 | 47 | 35 | 12 | 7.0 | 8.3 | 1.3 |
| 01-11 | 6 | 14.5 | 54 | 37 | 17 | 5.7 | 7.1 | 1.6 |
| Center 02 |  |  |  |  |  |  |  |  |
| 02-01 | 6 | 4.1 | 57 | 53 | 4 | 4.5 | 5.1 | 0.8 |
| 02-02 | 6 | 2.4 | 55 | 58 | -3 | 4.11 | 5.3 | 0.4 |
| 02-03 | 5 | 6.2 | 37 | 46 | -9 | 5.7 | 5.8 | 0.1 |
| 02-04 | 6 | 6.7 | 61 | 58 | 3 | 10.8 | 10.9 | 0.1 |
| 02-05 | 6 | 17.5 | 58 | 56 | 2 | 7.1 | 8.3 | 1.2 |
| 02-06 | 3 | 2.7 | 59 | 59 | ND | 4.6 | ND | ND |
| 02-07 | 6 | 1.8 | 52 | 55 | -3 | 5.1 | 6.0 | 0.11 |
| 02-08 | 6 | 4.2 | 49 | 42 | 7 | 5.1 | 6.1 | 1 |
| 02-09 | 5 | 7.7 | 45 | 46 | -1 | 5.1 | 5.7 | 0.6 |
| 02-10 | 3 | 5.1 | 72 | ND | ND | 4.8 | 4.8 | ND |
| 02-11 | 2 | ND | 8 | ND | ND | 2.5 | 2.5 | ND |

N.D.= not done

* total score
