# Supplementary material for: Intra-Erythrocyte Infusion of Dexamethasone Reduces Neurological Symptoms in Ataxia Teleangiectasia Patients: Results of a Phase 2 Trial
Source: Orphanet J Rare Dis. 2014 Jan 9;9:5. doi: 10.1186/1750-1172-9-5 (PMC3904207; doi:10.1186/1750-1172-9-5)
Supplement: Additional file 3: Figure S1 — Study disposition. [file 1750-1172-9-5-S3.docx]

Additional Figure 1 – Study disposition

screened

n = 26

enrolled

n = 22

screening failure

n = 4

CD4+ lymphocytes below the limit n = 3

concomitant disease

n = 1

premature terminations n = 4

completed study

n = 18

CD4+ lymphocytes below the limit n = 1

*(protocol violation)*

consent withdrawal

n = 1

dropouts due to AEs n = 2
