# Supplementary material for: Intra-Erythrocyte Infusion of Dexamethasone Reduces Neurological Symptoms in Ataxia Teleangiectasia Patients: Results of a Phase 2 Trial
Source: Orphanet J Rare Dis. 2014 Jan 9;9:5. doi: 10.1186/1750-1172-9-5 (PMC3904207; doi:10.1186/1750-1172-9-5)
Supplement: Additional file 5: Table S3 — ICARS and VABS scores in 4 patients treated for an adjunctive 19-month period as compared with 4 ICARS score matched controls. [file 1750-1172-9-5-S5.docx]

Additional Table 3. ICARS and VABS scores in 4 patients treated for an adjunctive 19-month period as compared with 4 ICARS score matched controls

| **Patient** | **ICARS score**  **V1** | **ICARS score**  **V7** | **ICARS score**  **19 mo** | **Kinetic Function** Subscale score **V1** | **Kinetic Function** Subscale score **V7** | **Kinetic Function** Subscale score **19 mo** | **VABS score**  **V1** | **VABS score**  **V7** | **VABS score**  **19 mo** |
| --- | --- | --- | --- | --- | --- | --- | --- | --- | --- |
| Extended treatment | | | | | | | | | |
| 02-01 | 57 | 53 | 45 | 24 | 21 | 19 | 4,5 | 5,1 | 5,2 |
| 02-02 | 55 | 58 | 51 | 22 | 21 | 19 | 4,11 | 5,3 | 5,7 |
| 02-05 | 58 | 56 | 43 | 23 | 21 | 13 | 7,1 | 8,3 | 13 |
| 02-08 | e49 | 42 | 38 | 23 | 23 | 15 | 5,1 | 6,1 | 8,5 |
| Interrupted treatment (at V7) | | | | | | | | | |
| 01-02 | 42 | 26 | 41 | 23 | 13 | 19 | 4,5 | 5,1 | 8,7 |
| 01-07 | 51 | 50 | 65 | 20 | 20 | 27 | 4,4 | 8,8 | 5,9 |
| 01-10 | 47 | 35 | 61 | 19 | 15 | 24 | 7,0 | 8,3 | 5,6 |
| 01-11 | 54 | 41 | 55 | 24 | 16 | 23 | 5,1 | 6,1 | 6,8 |
