# Supplementary material for: Intra-Erythrocyte Infusion of Dexamethasone Reduces Neurological Symptoms in Ataxia Teleangiectasia Patients: Results of a Phase 2 Trial
Source: Orphanet J Rare Dis. 2014 Jan 9;9:5. doi: 10.1186/1750-1172-9-5 (PMC3904207; doi:10.1186/1750-1172-9-5)
Supplement: Additional file 6: Table S4 — Values for Special Laboratory Parameters and adverse events in 4 patients treated for an adjunctive 19-month period as compared with 4 ICARS score matched controls. [file 1750-1172-9-5-S6.docx]

Additional Table 4. Values for special laboratory parameters and adverse events in 4 patients treated for an adjunctive 19-month

period as compared with 4 ICARS score matched controls .

| **EXTENDED TREATMENT (V7 TO 19 MONTHS)** | | | | | | | | | | | | | |
| --- | --- | --- | --- | --- | --- | --- | --- | --- | --- | --- | --- | --- | --- |
| **PARAMETERS** *(units)* | **Pz 02-01** | | | **Pz 02-02** | | | **Pz 02-05** | | | **Pz 02-08** | | | **normal ranges** |
|  | **V1** | **V7** | **V19 m** | **V1** | **V7** | **V19 m** | **V1** | **V7** | **V19 m** | **V1** | **V7** | **V19 m** |  |
| total cholesterol*(mmol/L)* | 4.64 | 4.43 | 4.20 | 4.17 | 3.74 | 3.59 | 3.43 | 3.10 | 2.76 | 5 | 4.87 | 5.10 | 2.84-5.3 |
| HDL cholesterol *(mmol/L)* | 1.28 | 1.17 | 1.28 | 1.56 | 1.46 | 1.56 | 1.12 | 1 | 1.12 | 1.30 | 1.43 | 1.30 | 0.6-2.05 |
| LDL cholesterol *(mmol/L)* | 3.21 | 2.87 | 2.97 | 2.38 | 1.87 | 2.05 | 1.89 | 1.76 | 1.30 | 3.30 | 3.12 | 3.46 | 1.17-3.63 |
| HbA1c *(%)* | 5.3 | 5.3 | 5.4 | 5.3 | 5.2 | 5.2 | 5.7 | 6 | 5.3 | 6 | 6 | 5.6 | 4.0-6.0 |
| CD4+ lymphocytes *(count/mm^3^)* | 278 | 470 | 267 | 243 | 230 | 264 | 679 | 681 | 686 | 276 | 304 | 373 | 410-1590 |
| α-fetoprotein *(μg/L)* | 102 | 114 | 97 | 388 | 364 | 383 | 87 | 77 | 96 | 118 | 123 | 109 | 0-5.0 |
| blood cortisol *(μg/dL)* | 8 | 16 | 15 | 8 | 11 | 12 | 6 | 7 | 15 | 8 | 12 | 11 | 2.4-22.9 |
| urinary cortisol *(μg/24h)* | ND | 28 | ND | ND | 72 | ND | 55.5 | 48.6 | ND | 63 | 25.6 | ND | 26.2-134.8 |
| **ADVERS EVENTS V19** | None | | | None | | | None | | | None | | |  |

| **INTERRUPED TREATMENT (V7)** | | | | | | | | | | | | | |
| --- | --- | --- | --- | --- | --- | --- | --- | --- | --- | --- | --- | --- | --- |
| **PARAMETERS** *(units)* | **Pz 01-02** | | | **Pz 01-07** | | | **Pz 01-10** | | | **Pz 01-11** | | | **normal ranges** |
|  | **V1** | **V7** | **V19 m** | **V1** | **V7** | **V19 m** | **V1** | **V7** | **V19 m** | **V1** | **V7** | **V19 m** |  |
| total cholesterol  *(mmol/L)* | 5.09 | 4.85 | 4.87 | 3.16 | 3.36 | 3.56 | 4.3 | 3.87 | 4.75 | 4.54 | 5.5 | 5.83 | 2.84-5.3 |
| HDL cholesterol *(mmol/L)* | 1.12 | 1.38 | 1.22 | 1.4 | 1.39 | 1.79 | 1.46 | 1.43 | 1.35 | 1.72 | 2.21 | 2.16 | 0.6-2.05 |
| LDL cholesterol *(mmol/L)* | 3.54 | 3.16 | 3.3 | 1.45 | 1.61 | 1.54 | 2,5 | 2.12 | 2.71 | 2.22 | 2.98 | 3.2 | 1.17-3.63 |
| HbA1c *(%)* | 5 | 4.9 | 5.1 | 4.9 | 4.9 | N.D. | 5.0 | 5.1 | 5.2 | 5.2 | 5.2 | 5.5 | 4.0-6.0 |
| CD4+ lymphocytes *(count/mm^3^)* | 315 | 509 | N.D. | 384 | 376 | N.D. | 482 | 481 | N.D. | 300 | 495 | N.D. | 410-1590 |
| α-fetoprotein *(μg/L)* | 88 | 130 | 127.0 | 86 | 91 | N.D. | 223 | 246 | N.D. | 433 | 471 | 263.0 | 0-5.0 |
| blood cortisol *(μg/dL)* | 27.4 | 6.3 | N.D. | 17.6 | 9 | N.D. | N.D. | 20.3 | N.D. | 9.1 | 9 | N.D. | 2.4-22.9 |
| urinary cortisol *(μg/24h)* | 44.3 | N.D. | N.D. | 43.8 | 88.3 | N.D. | N.D. | 17.8 | N.D. | 50.7 | 55.3 | N.D. | 26.2-134.8 |
| **ILLNESS IN 19 MONTHS** | bronchitis | | | none | | | Recurrent Infectious disease | | | none | | |  |

N.D.= not done
