# Supplementary material for: Intra-Erythrocyte Infusion of Dexamethasone Reduces Neurological Symptoms in Ataxia Teleangiectasia Patients: Results of a Phase 2 Trial
Source: Orphanet J Rare Dis. 2014 Jan 9;9:5. doi: 10.1186/1750-1172-9-5 (PMC3904207; doi:10.1186/1750-1172-9-5)
Supplement: Additional file 7: Table S5 — Changes from Baseline to the Final Visit for special laboratory parameters (ITT Population). [file 1750-1172-9-5-S7.docx]

*.*

Additional Table 5. Mean Values (SD) and Changes from Baseline to the Final Visit for Special Laboratory Parameters (ITT Population)

| **PARAMETERS** *(units)* | **mean (SD)** | | **absolute Δ (SD)** | **normal ranges** |
| --- | --- | --- | --- | --- |
|  | **V1** | **V7** |  |  |
| total cholesterol *(mmol/L)* | 4.3 (0.7) | 4.3 (0.8) | -0.006 (0.4) | 2.84-5.3 |
| HDL cholesterol *(mmol/L)* | 1.2 (0.3) | 1.3 (0.3) | 0.06 (0.2) | 0.6-2.05 |
| LDL cholesterol *(mmol/L)* | 2.7 (0.6) | 2.6 (0.7) | -0.12 (0.3) | 1.17-3.63 |
| HbA1c *(%)* | 5.2 (0.3) | 5.3 (0.3) | 0.1 (0.2) | 4.0-6.0 |
| CD4+ lymphocytes *(count/mm^3^)* | 379.7 (152.0) | 431.5 (147.2) | 51.8 (117.1) | 410-1590 |
| α-fetoprotein *(μg/L)* | 226.1 (161.8) | 234.6 (180.7) | 9.7 (34.2) | 0-5.0 |
| blood cortisol *(μg/dL)* | 15.9 (7.7) | 13.8 (6.4) | -2.9 (7.9) | 2.4-22.9 |
| urinary cortisol *(μg/24h)* | 39.9 (17.5) | 29.4 (20.3) | -11.9 (23.7) | 26.2-134.8 |

*Descriptive Text from CSR*

As observed for the standard laboratory parameters, the special laboratory parameters also showed minor fluctuations over the study period. α-fetoprotein values were abnormal (higher than normal range) at V1, and remained high throughout the study.

Patient # 01-08 showed increased serum cholesterol levels from the normal value recorded at baseline, which were reported as an AE, assessed as mild in intensity and considered related to the IMP.

Although there were three drop-outs related to a decrease in CD4+ lymphocytes count overall a mean increase of 51.8/mm^3^ (117.1) at V7 vs. V1 was observed [from 379.7±152.0/mm^3^ at V1 to 431.5±147.2/mm^3^ at V7]

The only two parameters which showed statistically significant changes between V1 and V7 were HbA1c *(p= 0.015)* and urinary cortisol *(p= 0.016)*. The mean change (increase) for HbA1c was 0.1% (0.2), while urinary cortisol decreased by 11.9±23.7 μg/24h The latter would indicate a possible effect of the IMP on the hypothalamic-pituitary-adrenal axis, but an evaluation of the variations of urinary cortisol as a function of the dose actually administered showed that some of the biggest changes (decreases) were seen in patients who received infusions with the lowest bag doses.
