# Supplementary material for: Intra-Erythrocyte Infusion of Dexamethasone Reduces Neurological Symptoms in Ataxia Teleangiectasia Patients: Results of a Phase 2 Trial
Source: Orphanet J Rare Dis. 2014 Jan 9;9:5. doi: 10.1186/1750-1172-9-5 (PMC3904207; doi:10.1186/1750-1172-9-5)
Supplement: Additional file 8: Table S6 — Group data for Vital and Physical Signs at V1 and V7 (ITT Population). [file 1750-1172-9-5-S8.docx]

Additional Table 6. Group data for Vital and Physical Signs at V1 and V7 (ITT Population)

| **PARAMETERS***(unit)* | **mean (SD)** | | **absolute Δ (SD)** |
| --- | --- | --- | --- |
|  | **V1** | **V7** |  |
| SBP*(mmHg)* | 99.4 (9.4) | 96.9 (10.3) | -4.1 (16.9) |
| DBP*(mmHg)* | 64.0 (8.8) | 61.1 (7.3) | -5.0 (2.1) |
| HR *(bpm)* | 89.7 (13.8) | 95.3 (10.3) | 2.1 (13.2) |

SBP: systolic blood pressure

DBP: diastolic blood pressure

HR: heart rate

|  |  |
| --- | --- |
